# Supplementary material for: Stop signals delay synchrony more for finger tapping than vocalization: a dual modality study of rhythmic synchronization in the stop signal task
Source: PeerJ. 2018 Jul 12;6:e5242. doi: 10.7717/peerj.5242 (PMC6046193; doi:10.7717/peerj.5242)
Supplement: Supplemental Information 1 [file peerj-06-5242-s001.zip › Raw data - for submission/Instructions.docx]

Each folder contains the data collected per participant. Within each folder, you will find 3 text files and a Matlab data file.

| Name of file | Description |
| --- | --- |
| ScrambledIndexArray.txt | This file stores the information of the randomisation procedure |
| StaircaseForStop_1.0.0.txt | This file shows the staircase for stop |
| Stop_1.0.0.txt | This file contains all the information about trials. It also includes the info of the file “StaircaseForStop_1.0.0.txt” |
| Data.mat | This is a Matlab data file that contains all the variables that were extracted from subsequent data analysis. |

Data was extracted from the file “Stop_1.0.0.txt”, so we proceed to explain what this file contains in the next table. It has 9 columns.

| Name of column | Description |
| --- | --- |
| run | It has numbers from 1 to 8, which indicates the block number. |
| trial_n | It shows the trial consecutive number in a block |
| FactorA | It has either number 1 or 2. 1 indicates a manual block and 2 shows a vocal block. |
| FactorB | It has numbers 1 to 3. 1 corresponds to stop trials. 2 and 3 corresponds to go trials. |
| FactorC | It has number 1 and 2. It was used for randomisation purposes. |
| TargItem | It has number 1 to 5. It was used for randomisation purposes. |
| SSD | This column shows the time at which the stop signal delay was presented within a trial. It started at 200 ms, which was 200 ms before a beep. It is important to note that the beep was placed at 400 ms within a trial. |
| RT | This column shows the go synchronisation responses (go-SR). It is estimated from the time the SSD was presented. To obtain an accurate measure of go-SR, we sum the columns for the SSD and the RT. For example, the first line of the P3_SM shows that the SSD was 200 and the RT was 185 (columns 1 and 2 respectively of Table 1 below). If we sum these two columns, we get 385ms. 385 ms represents the go-SR (column 3 of Table 1 below), which was the time at which a response was given within the trial. If we want to know at what time the response was given with respect to the beat onset, we subtract 400 ms from the go-SR (column 4 of Table 1 below). Column shows -15 ms, which says that the response was given 15 ms prior the beat.  Table. 1. Example P3_SM   \| SSD \| RT \| Go-SR  [SSD + RT] \| Asynchrony  [(SSD+RT) - 400] \| \| --- \| --- \| --- \| --- \| \| 200 \| 185 \| 385 \| 385-400=-15 \| |
| ACC | This column shows the accuracy.  2 means there was a go trial and a successful response  1 means there was a stop trial with a successful stop  0 means there was a stop trial with an unsuccessful stop  99 means there was a miss response. |
| Manual/Vocal | Two numbers 1 and 2. 1 means manual, 2 means vocal |
| Ignore/Stop | Two numbers 1 and 2. 1 means ignore, 2 means stop |
