# Supplementary material for: Stop signals delay synchrony more for finger tapping than vocalization: a dual modality study of rhythmic synchronization in the stop signal task
Source: PeerJ. 2018 Jul 12;6:e5242. doi: 10.7717/peerj.5242 (PMC6046193; doi:10.7717/peerj.5242)
Supplement: Supplemental Information 2 [file peerj-06-5242-s002.docx]

Comparison of reaction times from Presentation software against a Matlab code that detected the sound envelope of the voice signal

| RT obtained from Matlab code | RT obtained from Presentation Software |
| --- | --- |
| 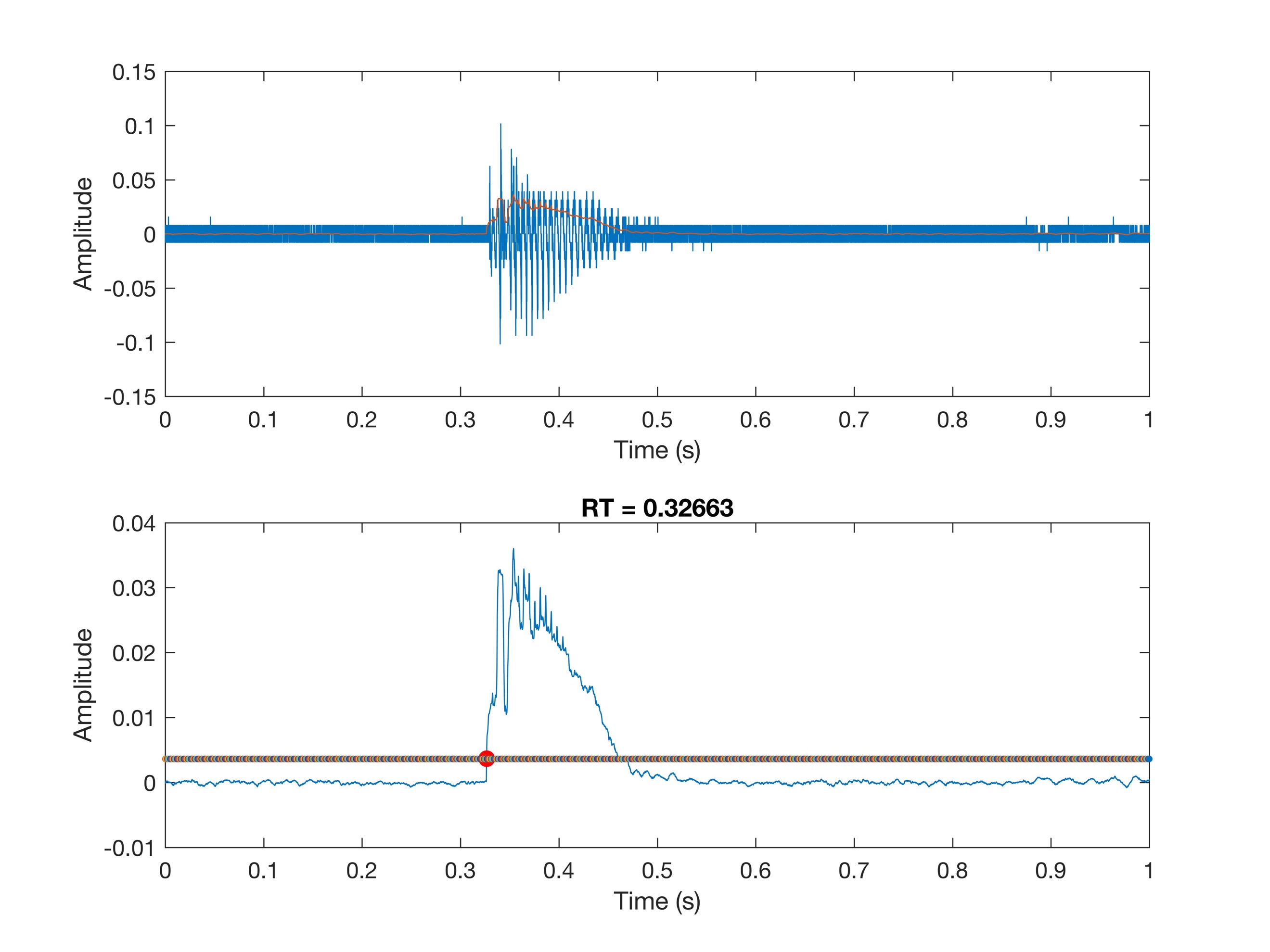  RT = 0.326 s | RT = 0.329 s |
| 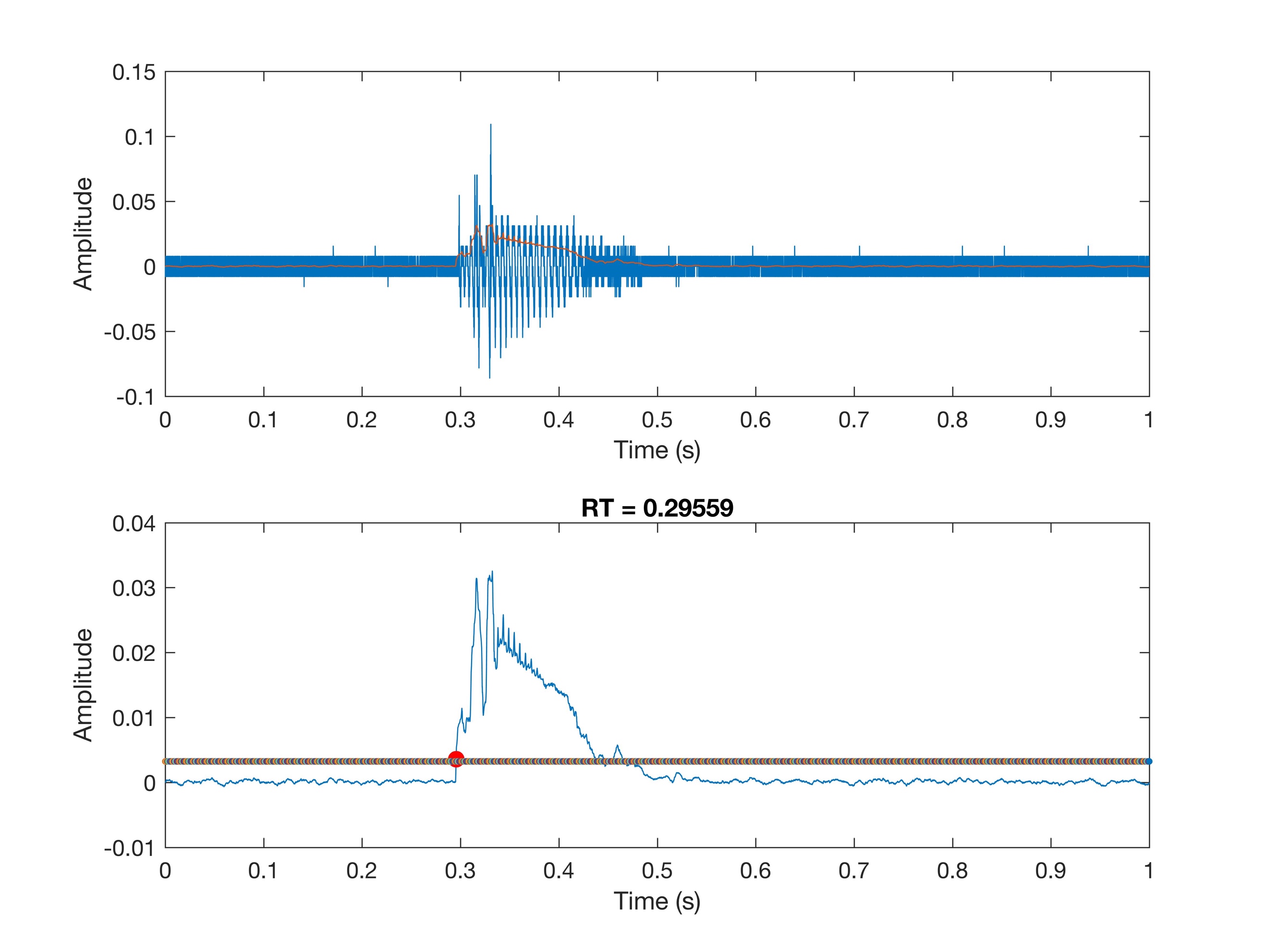  RT = 0.296 s | RT = 0.298 s |
| 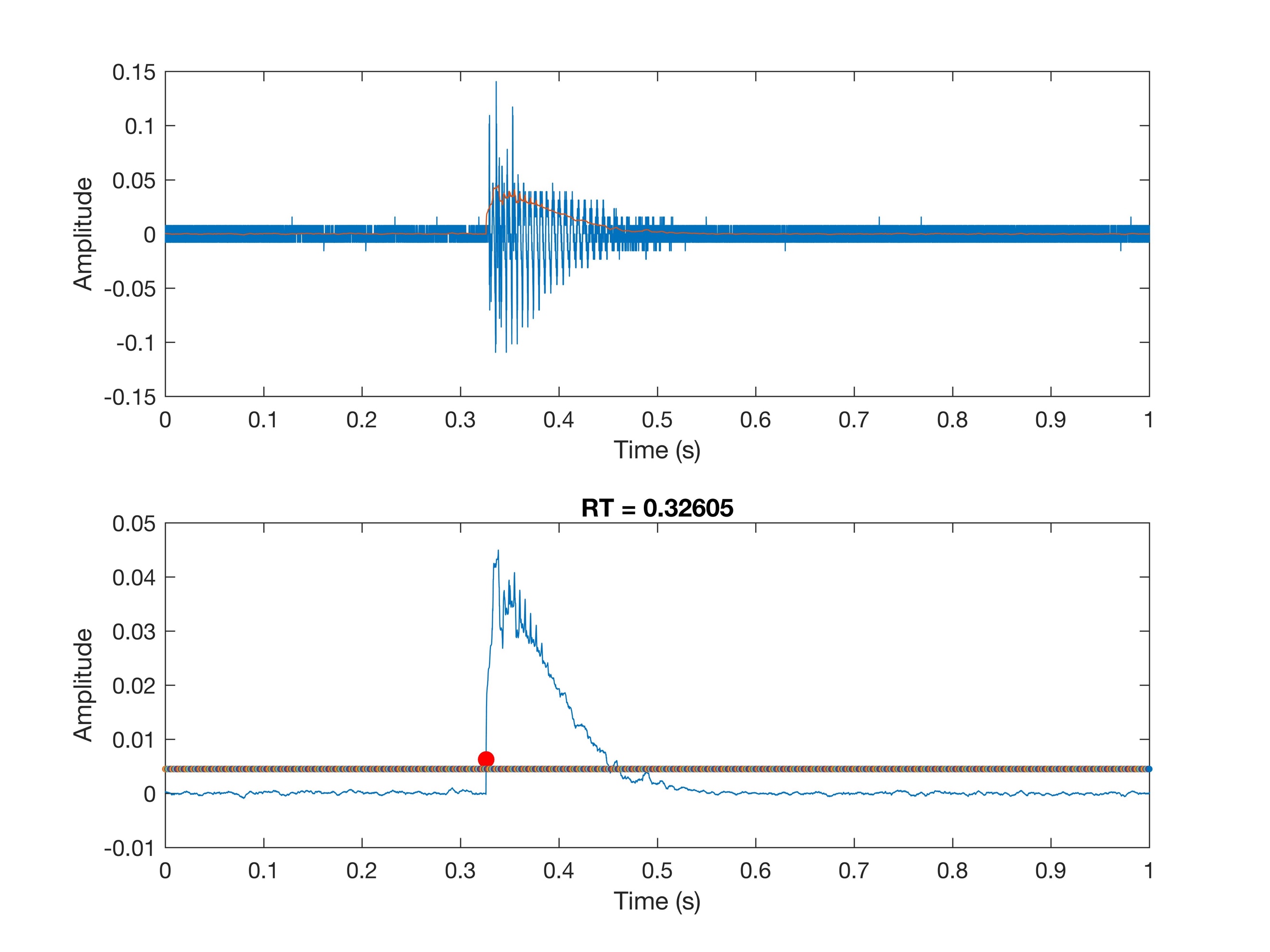  RT = 0.326 s | RT = 0. 329 s |
